# Posterior vitreous detachment and paravascular retinoschisis in highly myopic young patients detected by ultra-widefield OCT

**DOI:** 10.1038/s41598-021-96783-w

**Published:** 2021-08-30

**Authors:** Hiroyuki Takahashi, Noriko Nakao, Kosei Shinohara, Keigo Sugisawa, Kengo Uramoto, Tae Igarashi-Yokoi, Takeshi Yoshida, Kyoko Ohno-Matsui

**Affiliations:** Department of Ophthalmology and Visual Science, Tokyo Medical and Dental University (TMDU) Graduate School of Medical and Dental Sciences, 1-5-45 Yushima, Bunkyo-ku, Tokyo, 113-8519 Japan

**Keywords:** Eye manifestations, Eye diseases

## Abstract

The purpose of this study was to determine the relationship between a posterior vitreous detachment (PVD) and retinoschisis (RS) in 73 highly myopic (HM) young patients age 16.4 ± 6.9 years and 24 non-HM children age 8.4 ± 1.5 years. The presence of the paravascular retinal abnormalities was determined in the images obtained by a ultra-widefield OCT (UWF OCT) instrument with an image field of 23 × 20 mm. The results showed that a partial PVD was detected in 15 (21%) of the HM patients, and the number increased significantly with increasing age (*P* = 0.02). PVDs of any type were not found in the non-HM eyes. The number of microvascular folds also increased with age in the HM patients (*P* = 0.03). Medium-reflective columnar tissues were present between the detached vitreous and inner retinal surface in 4 (5%) eyes of the HM patients. Myopic RS was found in 3 (4%) HM patients in the paravascular area but not in the macular area. These results suggest that early partial PVD may play a role in pathological and proliferative vitreous changes of HM eyes. An intense vitreoretinal traction with bridging tissues may cause the various paravascular retinal abnormalities. In HM eyes, paravascular RS is already present at an early age which may progress to macular RS with aging.

## Introduction

Highly myopic (HM) patients develop a posterior vitreous detachment (PVD) earlier than non-highly myopic (non-HM) patients^1–4^, and various pathologies such as macular retinoschisis (RS), vitreomacular traction, and macular hole, are induced during the progression of the PVD^5,6^. In addition, vitreous surgeons often encounter remnants of the posterior vitreous cortex on the inner surface of the retina during vitrectomy. Thus, it is possible that the structure of the posterior vitreous can be altered in HM eyes at a young age before the retinal abnormalities associated with HM develop.

Imaging the vitreous has been difficult because of its transparency and mobility. With the advent of OCT, the early stages of PVDs in non-HM young adults were detected earlier and in more detail by conventional OCT in the paramacular areas in an earlier study^7^. Tsukahara et al. investigated 36 non-HM eyes whose age ranged 21–29 years and found that either paramacular PVD or perifoveal PVD developed in 34 eyes (94%). These results suggested that PVD initially occurred at paramacular area by the third decade of life. However, the incidence of PVDs at different ages has not been investigated in detail.

Ultra-widefield OCT (UWF OCT) is a swept source OCT (SS OCT) device equipped with high refractive index lens which enables clinicians to examine the posterior vitreous in a wide and deep space^8^. With this device, we have been able to examine the vitreal changes of HM eyes of elderly patients, and it was noted that the presence of paravascular vitreal adhesions were important for the development of myopic macular retinoschisis (RS)^9^. However, a detailed examination of the chronological development of myopic RS has not been determined.

Thus, the purpose of this study was to determine the presence and location of PVDs and myopic RS in young patients using UWF-OCT. The incidence of PVD and paravascular abnormalities, and the effect of aging were also investigated in young HM patients.

## Methods

### Patients

This was a retrospective, single-center, observational case series. We enrolled HM patients of the Advanced Clinical Center for Myopia at the Tokyo Medical and Dental University between February 2017 and July 2018. To be eligible for participation, patients had to be HM and be from 6 to 30-years-of-age. HM was defined as a myopic refractive error (spherical equivalent) of > 6.0 diopters or an axial length > 26.0 mm. To focus on children and adolescents who were definitely HM, HM was defined for patients under 20-years-of-age the same as patients over 20-years-of-age. The exclusion criteria were patients with uveitis, rhegmatogenous retinal detachment, and congenital vitreoretinal abnormalities. The HM patients were divided into ages 6–12 years as HM children, 13–19 years as HM adolescents, and 20–30 years as HM young adults. Six- to 12-year-old children with myopic refractive error < 4.0 diopters and axial length < 26.0 mm were enrolled between July and December 2020 and served as non-HM controls. One eye of each pair HM and non-HM eyes was randomly selected for the statistical analyses.

The protocol used in this study was approved by the Ethics Committee of the Tokyo Medical and Dental University, and it conformed to the tenets of the Declaration of Helsinki. Adult patients provided a signed written informed consent, or a written informed consent was obtained from parents of the minor patients involved in the study. Approval was obtained from all patients to publish the retinal images included in this article.

The medical records were reviewed, and the values of the ocular parameters, age, and sex of the patients were collected. All of the participants underwent comprehensive ophthalmic examinations including dilated stereoscopic fundus examinations, measurement of the refractive error with an autorefractor (KR7100P; Topcon, Tokyo, Japan), measurement of the axial length (IOL Master; Carl Zeiss Meditec, Jana, Germany), and ultra-widefield fundus imaging with the Optos 200Tx scanning laser ophthalmoscopy (Optos PLC, Dunfermline, United Kingdom).

### Ultra-widefield swept-source OCT (UWF-OCT)

All patients underwent ultra-widefield imaging of the posterior fundi with an image size of 23 mm in the horizontal direction and 20 mm in the vertical direction with a prototype UWF OCT device. The UWF OCT system was based on a swept source laser with an A-scan repetition rate of 100,000 Hz (Canon Corp, Tokyo, Japan). Cross-sectional scans were centered on the fovea, and images of the posterior fundus with an angle of 80 degrees were obtained in less than 4 s.

A posterior staphyloma was defined as an outpouching of the posterior segment of the globe with a radius of curvature shorter than that of the surrounding orbital wall^10^. The presence of a posterior staphyloma was determined in the UWF-OCT images by identifying the staphyloma edges by three characteristic features; a gradual thinning of the choroid from the periphery toward the staphyloma edge, a gradual re-thickening of the choroid in a direction toward the posterior pole, and an inward protrusion of the sclera at the edge of the staphyloma^11^.

The posterior vitreal surface was defined as a traceable line extending above the inner retinal surface. The presence of a PVD was also determined in the UWF-OCT images by two masked investigators (HT and KOM). When the decision was not the same, the final decision was made based on the discussion between two graders. Eyes whose PVD were symmetrical with respect to the fovea were defined as ‘symmetrical PVD’, whereas a PVD that was asymmetrical with respect to the fovea was defined as ‘asymmetrical PVDs’. In eyes with a PVD, the presence of granular and columnar tissues between the detached vitreous cortex and the inner retinal surface was also determined.

The diagnosis of myopic RS was based on the presence of a splitting of the outer retinal layer in the UWF-OCT images^12^. The incidence of myopic RS was determined in the macular and paravascular areas. A macular RS was defined as a myopic RS within the central retinal field with diameter of 6 mm. A paravascular RS was defined as a myopic RS developing around retinal vessels outside the macular area. In addition, the presence of paravascular lesions such as paravascular retinal cysts, vascular microfolds, and paravascular lamellar holes, were also determined by the same two masked investigators (HT and KOM).

### Statistical analyses

Categorical variables are expressed as numbers and percentages, and the significance of their difference between two groups were determined by Fisher’s exact tests. Quantitative data were compared using unpaired *t* tests after their normal distributions were confirmed by Shapiro–Wilk tests. Mann–Whitney U tests were used for nonparametric datasets. All tests were two-sided. Cohen’s kappa values were used to evaluate the inter-grader reliability for detection of PVD. All of the analyses were performed with SPSS version 24.0 software (SPSS, Chicago Illinois, USA). A *P* value < 0.05 was considered to be statistically significant.

## Results

### Patient demographics

A total of 103 patients whose ages ranged from 6 to 30 years participated in the study, and all were examined by UWF-OCT. Four patients were excluded because of the presence of uveitis, and 2 patients were excluded because of the presence of a rhegmatogenous retinal detachment. In the end, 73 eyes of 73 HM patients and 24 eyes of 24 non-HM patients who met the criteria for inclusion were studied.

The clinical characteristics of the 73 HM eyes (73 patients) and 24 non-HM eyes (24 patients) are summarized in Table 1. Among the 73 HM patients, 27 were children, 22 were adolescents, and 24 were young adults. A comparison between the HM children and non-HM children showed that there was no significant difference in the mean age between the two groups (8.4 ± 1.5 years-old in non-HM and 9.4 ± 1.9 years-old in HM, *P* = 0.06). The mean axial length was significantly longer in the eyes of HM children (26.8 ± 0.9 mm) than that in eyes of non-HM children (24.6 ± 0.8 mm, *P* < 0.001). Posterior staphylomas were present in 7 (10%) of the 73 HM young patients but not in any of the non-HM patients.

**Table 1:** Clinical characteristics of young patients with and without high myopia. *HM* high myopia, *SD* standard deviation.

	Non-HM children	HM		
		Children	Adolescents	Young adults
No. of eyes (patients)	24 (24)	27 (27)	22 (22)	24 (24)
**Age (years)**				
Mean ± SD	8.4 ± 1.5	9.4 ± 1.9	15.0 ± 2.0	24.9 ± 2.9
Range	6 to 12	6 to 12	13 to 19	20 to 30
Male, no (%)	15 (63)	15 (56)	12 (55)	11 (46)
**Refractive error (diopters)**				
Mean ± SD	− 2.4 ± 0.8	− 8.5 ± 2.0	− 10.8 ± 3.3	− 11.3 ± 4.4
Range	− 0.9 to − 3.8	− 6.0 to − 14.8	− 6.5 to − 17.5	− 6.2 to − 21
**Axial length (mm)**				
Mean ± SD	24.6 ± 0.8	26.8 ± 0.9	28.2 ± 1.5	28.8 ± 1.5
Range	23.2 to 25.5	26.1 to 29.2	26.2 to 32.0	26.2 to 33.9
Posterior staphyloma, no (%)	0 (0)	2 (7)	2 (9)	3 (13)

In the diagnosis of PVD in the UWF OCT images, an agreement of both graders was made for 74 eyes (76%). The concordance between the two graders was considered to be good with a Cohen’s kappa value of 0.64 (95% confidence interval 0.54–0.74). The final diagnosis was made for all eyes based on the discussion between the two graders.

### Posterior vitreous detachment and paravascular retinoschisis in non-highly myopic and highly myopic children

**Figure 1:**
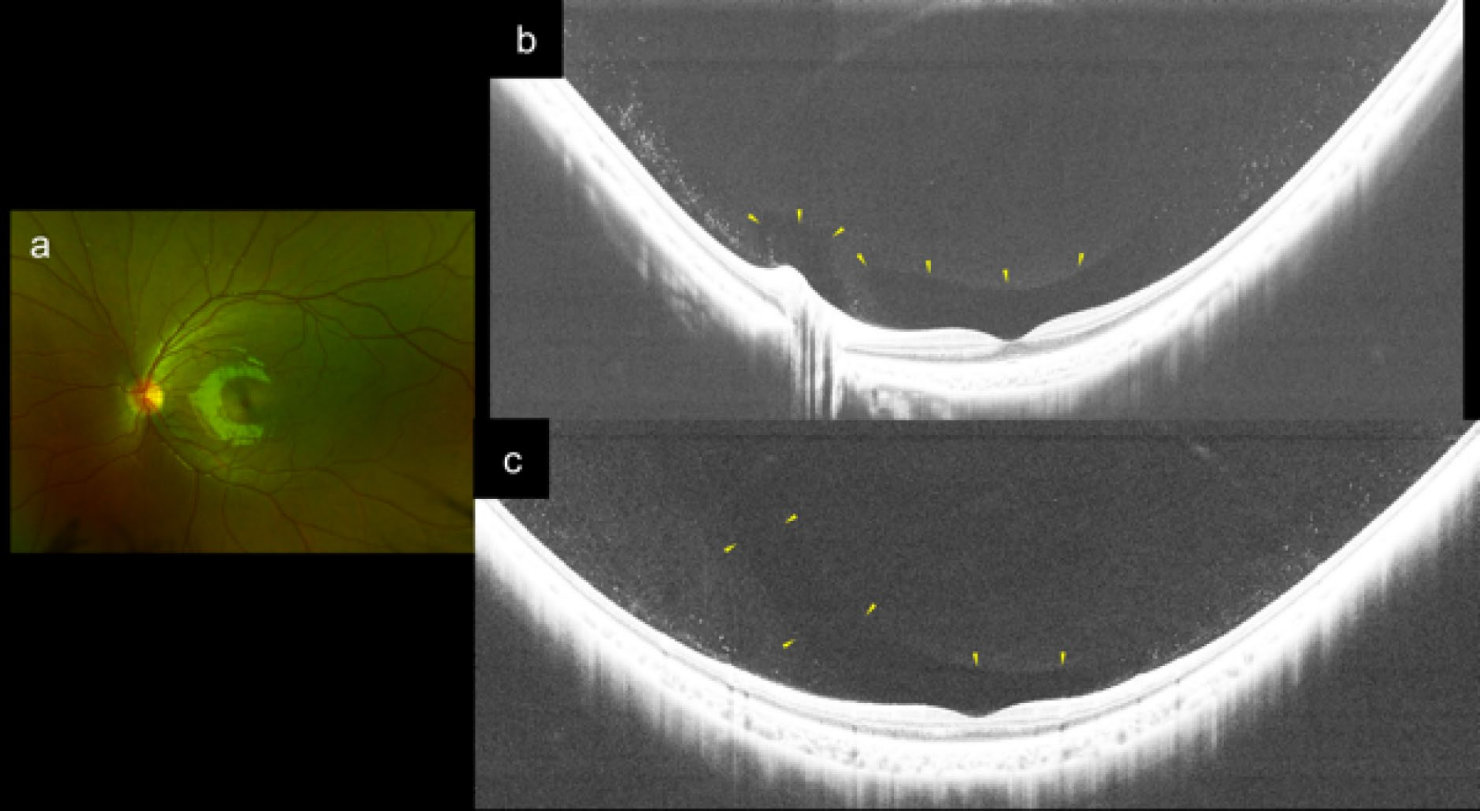
Findings in the posterior vitreous of an 8-year-old boy with moderate myopia and a refractive error of − 3.75 diopters and an axial length of 25.16 mm. (**a**) Fundus photograph of the left eye. (**b**) Brightened horizontal UWF OCT image showing that Cloguet’s canal and precortical posterior vitreous pocket (PPVP) are present above the inner retinal surface (yellow arrowheads) and medium reflective granular opacities are present in posterior vitreous cortex. (**c**) Brightened vertical UWF-OCT image showing that the superior edge of the PPVP extends anteriorly (yellow arrowheads).

**Figure 2:**
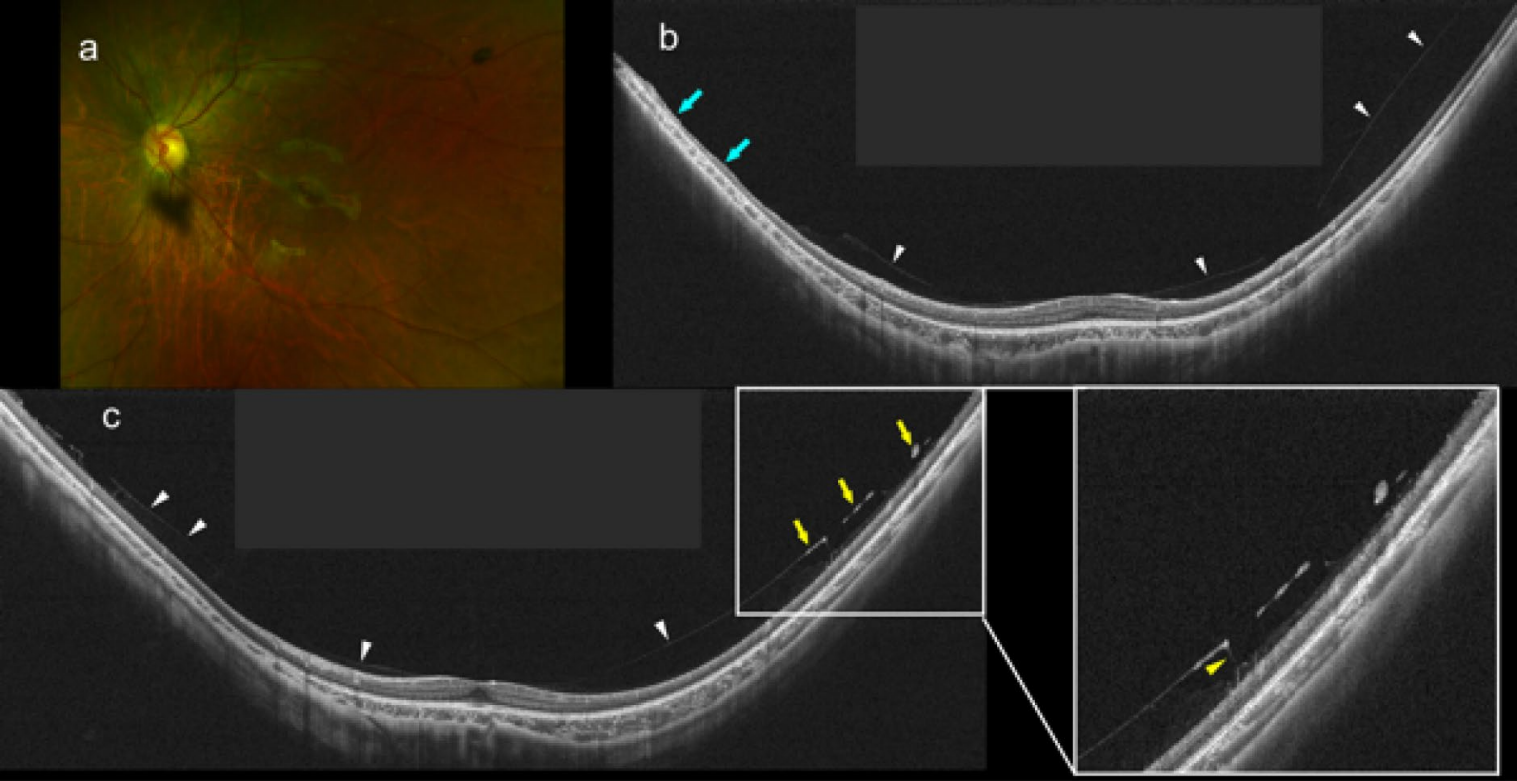
Partial posterior vitreous detachment in an 8-year-old boy with high myopia (HM). (**a**) Fundus photograph of left eye with a refractive error of − 10.5 diopters and an axial length of 26.8 mm. Tessellated fundus can be seen around the optic disc. (**b**) Vertical UWF OCT image showing asymmetrical perifoveal posterior vitreous detachment (white arrowheads). Posterior vitreous cortex is not seen at the superior paramacular area, and the retinal layers are more disorganized at the same area compared to other areas (light blue arrows). (**c**) Oblique UWF OCT image showing perifoveal posterior vitreous detachment (white arrowheads). Posterior vitreous cortex has a higher reflectivity at the paravascular area than in the other areas (yellow arrows). Medium reflective columnar and granular tissues are seen between the posterior vitreal surface and the inner retinal surface (yellow arrowheads).

None of the 24 eyes of the non-HM children had a partial PVD. The posterior vitreous had a Cloquet’s canal and the precortical posterior vitreous pockets were seen as hyporeflective spaces in these non-HM eyes (Fig. 1). Among the 27 eyes of HM children, partial PVDs were seen in 2 eyes (7.4%). Of these 2 eyes, one HM eye had medium-reflective tissues located between the detached vitreous and inner retinal surface (Fig. 2). The posterior vitreal surface had a relatively higher reflectivity and was thicker than that in the other areas around the site where this bridging tissue attached to the posterior vitreous (Fig. 2). A myopic RS was seen at the paravascular area in one HM eye (4%), but not in the non-HM eyes (Table 2).

**Table 2:** Comparisons of UWF-OCT findings between highly myopic and non-highly myopic children, and between different age groups of highly myopic patients.

	Children		*P* value	HM			*P* value	
	No-HM	HM		Children	Adolescents	Young Adults		
No. of eyes	24	27		27	22	24		
Partial PVD, no. (%)	0 (0)	2 (7)	0.49*	2 (7)	4 (18)	9 (38)		0.02^†^
Symmetrical PVD, no. (%)	0 (0)	1 (4)	> 0.99*	1 (4)	4 (18)	5 (21)		0.06^†^
Asymmetrical PVD, no. (%)	0 (0)	1 (4)	> 0.99*	1 (4)	0 (0)	4 (17)		0.16^†^
Columnar tissues between detached vitreous and inner retinal surface, no. (%)	0 (0)	1 (4)	> 0.99*	1 (4)	1 (5)	2 (8)		0.73^†^
Granular tissues between detached vitreous and inner retinal surface, no. (%)	0 (0)	2 (7)	0.49^a^	2 (7)	1 (5)	2 (8)		0.79^†^
Epiretinal membrane, no. (%)	0 (0)	0 (0)	N.A	0 (0)	0 (0)	0 (0)		N.A
Retinoschisis, no. (%)	0 (0)	1 (4)	> 0.99*	1 (4)	0 (0)	2 (8)		0.29^†^
Macular retinoschisis, no. (%)	0 (0)	0 (0)	N.A	0 (0)	0 (0)	0 (0)		N.A
Paravascular retinoschisis, no. (%)	0 (0)	1 (4)	> 0.99*	1 (4)	0 (0)	2 (8)		0.29^†^
Paravascular retinal cysts, no. (%)	0 (0)	2 (7)	0.49*	2 (7)	1 (5)	4 (17)		0.25^†^
Vascular microfolds, no. (%)	0 (0)	0 (0)	N.A	0 (0)	1 (5)	4 (17)		0.03^†^
Paravascular lamellar hole, no. (%)	0 (0)	0 (0)	N.A	0 (0)	1 (5)	2 (8)		0.26^†^

*HM* high myopia, *PVD* posterior vitreous detachment, *N.A* not applicable.

*Fisher’s exact probability test.

^†^Chi square test.

### Posterior vitreous detachment in highly myopic adolescents

**Figure 3:**
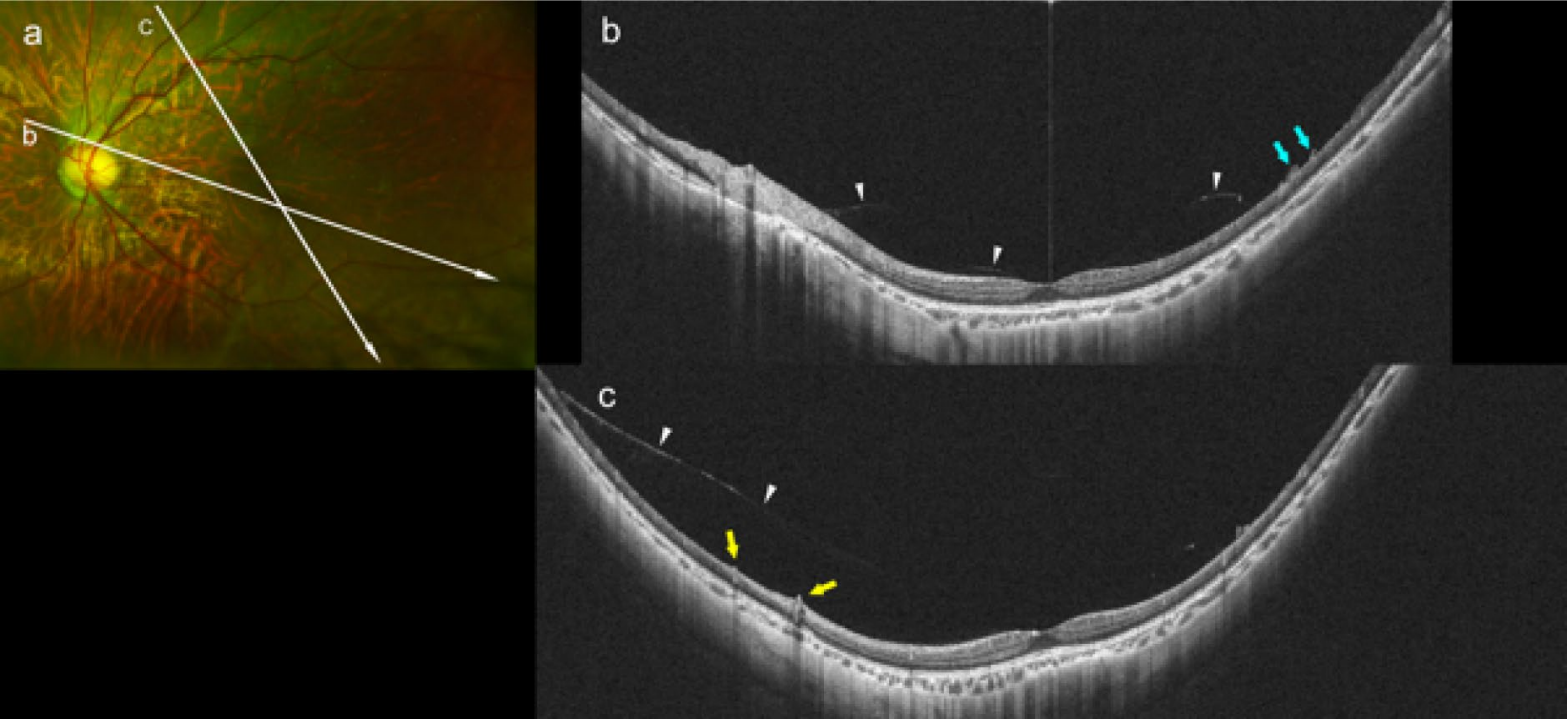
Asymmetrical posterior vitreous detachment in a 13-year-old girl with high myopia (HM). (**a**) Fundus photograph of the left eye with a refractive error of − 11.6 diopters and an axial length of 27.06 mm. Peripapillary diffuse choroidal atrophy can be seen. Arrows show the lines scanned by the ultra-widefield (UWF) OCT device. (**b**) Oblique UWF-OCT image showing a perifoveal posterior vitreous detachment (PVD; white arrowheads). Medium reflective tissues are present on the retinal surface at the area inferior to the fovea (light blue arrowheads). (**c**) Oblique UWF-OCT image showing that the perifoveal PVD extends in the superior direction asymmetrically (arrowheads). Microvascular folds are seen on the retinal surface at the area of the PVD (yellow arrows).

Partial PVDs were seen in 4 of the 22 eyes (18%) among the HM adolescents. In these 4 eyes, medium-reflective tissues were present on the relatively thinner retinal surface (Fig. 3a,b), and vascular microfolds with and without paravascular retinal cysts were also seen (Fig. 3a,c). None of the eyes had a myopic RS (Table 2).

### Posterior vitreous detachment and paravascular retinoschisis in highly myopic young adults

**Figure 4:**
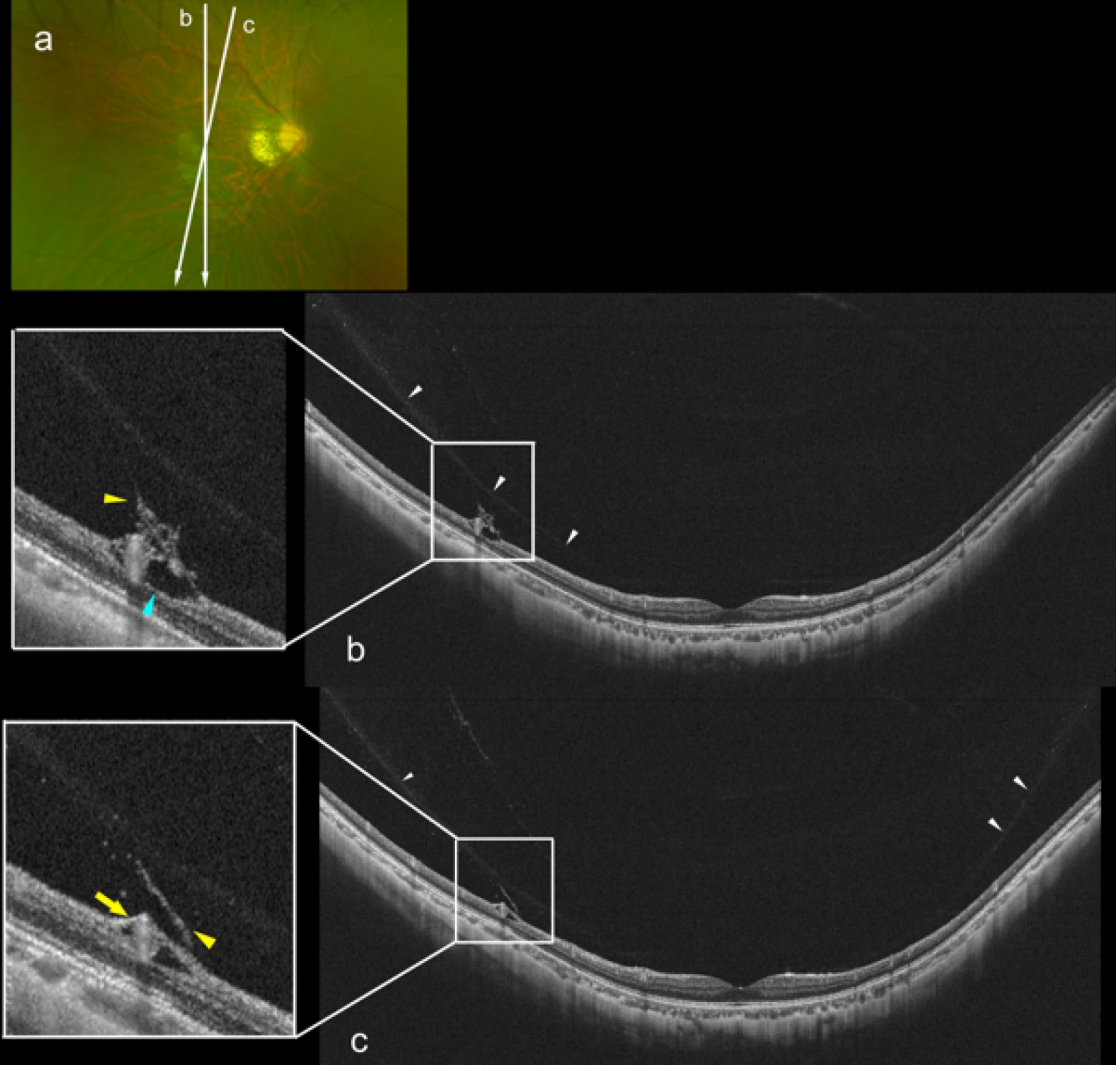
Posterior vitreous detachment (PVD) with bridging tissue between detached vitreous and retinal vessel in a 21-year-old man with high myopia (HM). (**a**) Fundus photograph of the right eye with a refractive error of − 9.3 diopters and an axial length of 28.25 mm. Tessellated fundus can be seen and a myopic peripapillary conus is present. Arrows show the lines scanned by the ultra-widefield (UWF) OCT device. (**b**) Vertical UWF OCT image showing a paramacular PVD at the area superior to the macula (white arrowhead). Medium reflective bridging tissue can be seen connecting the posterior vitreous surface and retinal vessels (yellow arrowhead). At the site of the adhesion, the retinal vessels are lifted anteriorly. Paravascular retinal cyst are also present (light blue arrowhead). (**c**) Oblique UWF OCT image showing a symmetrical PVD (white arrowheads). Medium reflective bridging tissue connects the posterior vitreous and retinal vessel (yellow arrowhead), and vascular microfolds are present at the site of the adhesion (yellow arrow).

**Figure 5:**
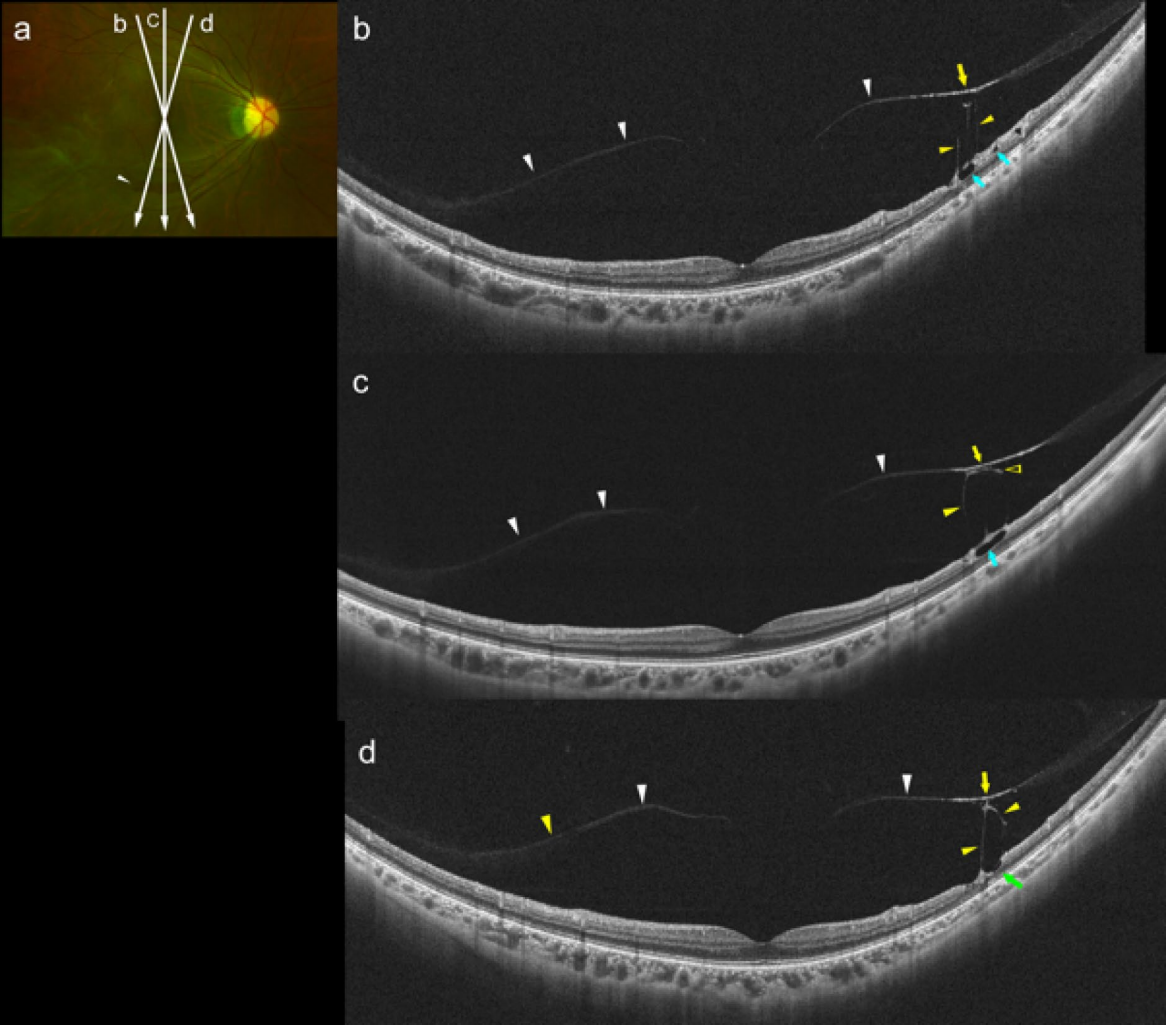
Findings in a 27-year-old woman with high myopia (HM) showing vitreous cortex splitting and bridging tissue between the detached vitreous and retinal vessels. (**a**) Fundus photograph of the right eye with a refractive error of − 8.4 diopters and axial length of 26.23 mm. Arrows show the lines scanned by the ultra-widefield (UWF) OCT device. Inner retinal hemorrhage is seen around the retinal vein inferior to the macular area (white arrow). (**b**) Oblique UWF OCT image showing a partial posterior vitreous detachment (white arrowheads). Part of the posterior vitreous cortex is connected to the inner retinal surface by bridging tissues with higher reflectivity (yellow arrow). Bridging tissues aligned perpendicularly from the inner retinal surface (yellow arrowheads). Paravascular retinal cysts are seen at the site of the adhesions (light blue arrows). (**c**) Vertical UWF OCT image showing posterior vitreous detachment (white arrowheads). Part of the vitreal surface shows adhesions to bridging tissues (yellow arrowheads) and higher reflectivity (yellow arrow). Medium reflective tissue bridges between the posterior vitreal surface with relatively higher reflectivity and inner retinal surface accompanied with retinal cyst (yellow arrows). An operculum-like tissue adheres to the posterior vitreous surface (open yellow arrow) and large paravascular cyst can be seen (light blue arrow). (**d**) Adjacent oblique UWF OCT image showing posterior vitreous detachment (white arrowheads), and a part of the vitreal surface shows adhesions to bridging tissues (yellow arrowheads) and a higher reflectivity (yellow arrow). Paravascular lamellar hole is present at the site of an adhesion (light green arrow).

Partial PVDs were seen in 9 of 24 eyes (38%) of the HM young adults. Medium-reflective bridging tissues were seen adhered to the posterior vitreal surface, and they were accompanied by medium-reflective granular opacities in 2 eyes (Fig. 4). The posterior vitreal surface had a relatively higher reflectivity than the other areas at the site of the adhesion (Fig. 5). In addition, paravascular retinal cysts and paravascular lamellar holes were found at the sites where the bridging tissues were adhered to the inner retinal surface (Fig. 5). Paravascular RS was seen in 2 eyes (8%) and both had paravascular retinal cysts (Table 2).

### Increase of partial PVD and paravascular abnormalities in different age groups of highly myopic patients

The incidence of partial PVDs and vascular microfolds increased with age, and there was a significant difference in the incidence between the age groups of HM patients (*P* = 0.02 and *P* = 0.03 respectively, Table 2). On the other hand, the incidences of paravascular retinal cysts and paravascular lamellar holes were not associated with age in the HM patients. PVDs developed asymmetrically rather than symmetrically in HM patients. Among 10 HM eyes with asymmetrical PVDs, a PVD was present in the superior quadrant in 6 eyes and in the inferior quadrant in the other 4 eyes.

### Relationship between paravascular retinoschisis and other paravascular abnormalities in HM patients

**Figure 6:**
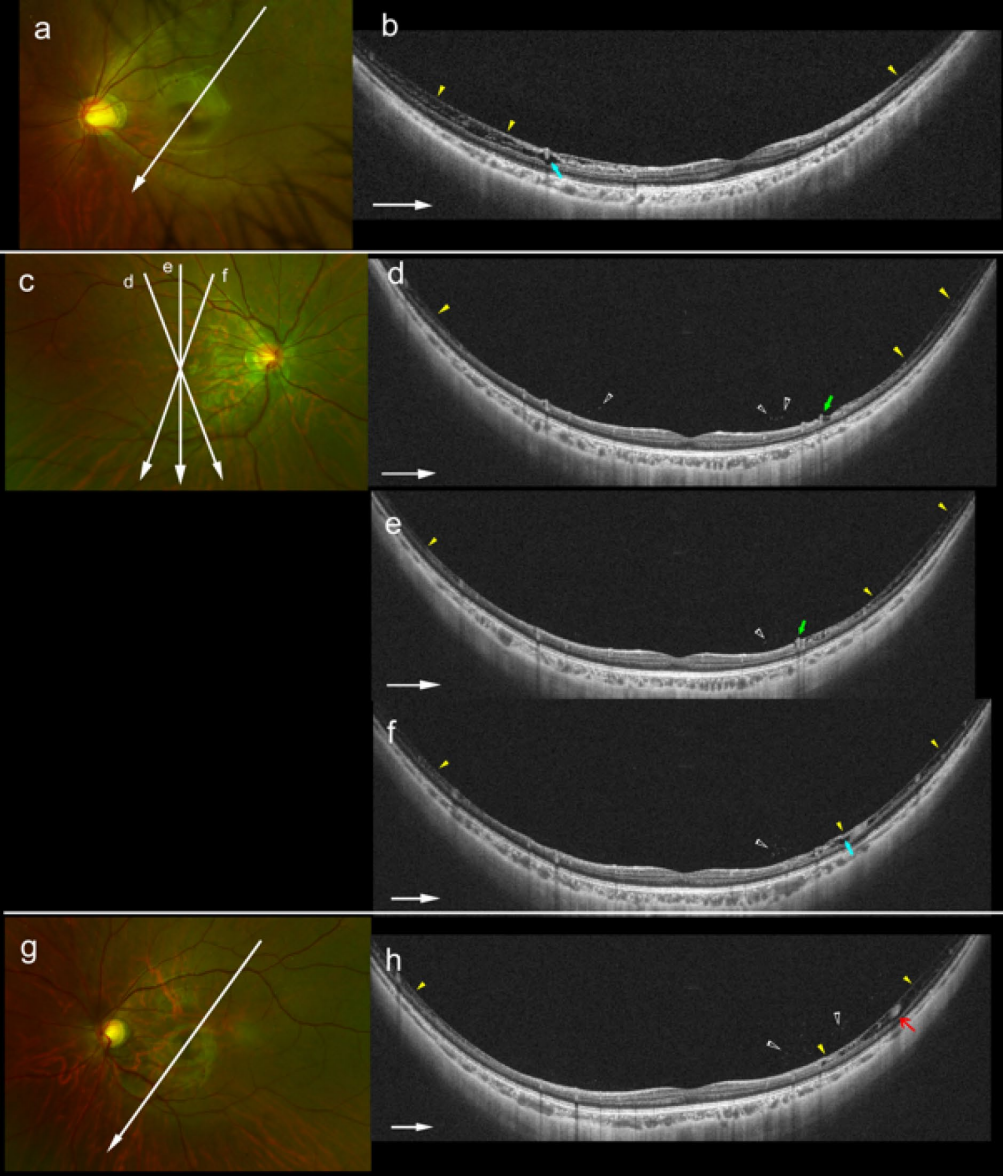
Images showing paravascular retinoschisis (RS) in young highly myopic (HM) eyes. (**a**) Left fundus of a 9-year-old girl with high myopia and refractive error of − 8.0 diopters (D). Arrow shows the line scanned by the ultra-widefield (UWF) OCT. (**b**) UWF-OCT image showing inner RS extending from the paramacular area to the peripheral area on the temporal-superior side of the fovea (arrowheads). Paravascular retinal cysts are present within the area of the RS (arrow). (**c**) Right fundus of a 21-year-old woman with HM and refractive error of − 8.6 D: Arrows show the lines scanned by the UWF-OCT. (**d**) and (**e**) Sequential radial sections of UWF-OCT images showing inner RS extending from the paramacular area to the peripheral area (yellow arrowheads). Intravitreal granular opacities are seen in the paravascular vitreous (open arrowheads). Retinal vessels are lifted up anteriorly (green arrows). (**f**) Paravascular retinal cysts are seen within the area of RS (light blue arrow). (**g**) Left fundus of the same patients as in (**c**). Refractive error is − 8.9 D in the left eye. (**h**) Oblique UWF-OCT image showing inner RS extending from the paramacular area to the peripheral area (yellow arrowheads). Intravitreal opacities are present within the paravascular vitreous (open arrowheads). Retinal vessel is surrounded by paravascular RS (red arrow).

A retinochisis was seen in one HM child and 2 HM young adults (Fig. 6). None of the 3 eyes with myopic RS had a PVD, and the myopic RS in these 3 eyes was a paravascular RS. None of the eyes had macular RS. HM eyes with retinoschisis also had paravascular retinal cysts and vascular microfolds more frequently than HM eyes without myopic RS (*P* = 0.001 and *P* = 0.01, respectively, Table 3). On the other hand, there was no difference in the incidence of PVD and paravascular lamellar holes between HM eyes with and without myopic RS.

**Table 3:** Comparison of UWF-OCT findings of vitreous and retina in highly myopic young patients with and without retinoschisis.

	Retinoschisis		*P* value
	Present	Absent	
No. of eyes (patients)	3 (3)	70 (70)	
Partial posterior vitreous detachment, no. (%)	0 (0)	13 (19)	> 0.99*
Bridging tissue between detached vitreous surface and inner retinal surface, no. (%)	0 (0)	4 (6)	> 0.99*
Epiretinal membrane, no. (%)	0 (0)	0 (0)	
Paravascular retinal cysts, no. (%)	3 (100)	4 (6)	0.001*
Vascular microfolds, no. (%)	2 (67)	3 (4)	0.01*
Paravascular lamellar holes, no. (%)	0 (0)	3 (4)	> 0.99*

*Fisher’s exact probability test.

## Discussion

The UWF-OCT images enabled us to examine the posterior vitreous in greater detail in children as well as young adults. Earlier studies reported that both conventional spectral domain OCT or SS OCT can obtain images of the vitreous but the scanned area was limited to the macular area. To overcome this limitation, the imaging fields of OCT devices have been extended^13^. In addition, it has been difficult to obtain images of the vitreous in the area outside the macula especially in children because children cannot maintain a steady fixation which is required during the examinations. UWF-OCT can obtain vitreal images that are 23 (H) × 20 (V) mm in just 4 s which has been very helpful in examining children.

Among the eyes of children ages 6–12 years, partial PVDs were seen in 7% of the HM eyes but none in the non-HM eyes. Previous studies showed that PVDs developed earlier in HM than non-HM young adults^1–4^. Our results showed that the PVD might start to develop much earlier in HM eyes than reported and even during childhood.

Of the 15 HM eyes with a partial PVD, 10 eyes had an asymmetrical partial PVD. The asymmetrical PVDs developed in the vertical direction in all of the 10 eyes, and 6 eyes had a superior partial PVD and the other 4 eyes had an inferior partial PVD. Tsukahara et al. investigated the posterior vitreous of 98 healthy normal adults and reported that a PVD began at the superior quadrant more frequently^7^. However, it has not been determined how and why PVDs begin at the superior quadrant. We recently reported that fluid cisternae were commonly seen within the vitreous gel that extended towards the periphery in the superior quadrant in the three-dimensional OCT images obtained by AI-applied segmentation^14^. Thus, the decrease of the density of the superior vitreous gel might make the vitreous more fragile structurally and may induce earlier PVDs in these locations. Takahashi et al. examined 167 eyes of HM patients whose ages were > 50 years and reported asymmetrical PVDs were commonly seen in HM patients^8^. Different from the results in the current study, Takahashi et al. showed that asymmetrical PVDs were seen commonly either at the inferior quadrant or at the area where the sclera protruded most posteriorly, usually the temporal quadrant, in HM eyes of older patients^8^. These findings suggested that although asymmetrical PVDs may be common in HM patients in general, they appeared to be distributed differently according to age, and different factors may be involved in the pathogenesis of partial PVDs. Scleral deformities such as posterior staphylomas increase during adulthood in HM patients, and the progression of PVDs might differ between young and old HM patients.

Tissues bridging the posterior surface of vitreous and inner retinal surface were seen in 4 HM eyes. Mojana et al. reported similar bridging tissues connecting the posterior vitreous surface and the retinal vessels during the development of PVDs in adult eyes, however the relationship between HM and bridging tissue was not analyzed^15^. At the site of the adhesions, the reflectivity of the vitreous cortex become brighter than the other area, and lamellar structures of vitreous cortex appeared to merge as a thick vitreous membrane (Fig. 5). Thick posterior vitreous membranes in young HM patients might induce light scattering and appear as floaters in these HM young patients as is often seen in eyes without a complete PVD.

Large hyporeflective cystic lesions were seen next to the vessels in the inner retinal layer at the sites of the paravascular vitreal adhesions (Figs. 4 and 5). These findings suggested that abnormal paravascular vitreal adhesions may cause traction on the retinal vessels as the partial PVD progressed which then forms the paravascular abnormalities. In addition, granular tissues were present between the detached vitreous and the inner retinal surface (Figs. 4 and 5). We suggest that such granular tissues might be the migration of deteriorated perivascular cells, such as glia cells, into the vitreous cortex during the progress of PVD. A migration of paravascular glial cells into the vitreous of HM children or young adults may cause pathological and proliferative changes in vitreous.

Our results showed that 7% of HM children, 18% of HM adolescents, and 38% of HM young adults had partial PVD, and the differences between the age groups were significant in the HM patients. Among the paravacular retinal abnormalities, the incidence of vascular microfolds was also significantly correlated with the age of the patients, but the incidence of paravascular RS, paravascular retinal cysts, and paravascular lamellar holes were not. Vascular microfolds were reported to be present in vitrectomized and non-vitrectomized eyes of older HM adults in previous studies^16,17^. Accordingly, we suggest that earlier PVD formation might lead to traction on the inner retinal surface and result in the development of microvascular folds in young HM patients. The results of this study showed that vascular microfolds occurred at a much younger age than reported.

The exact cause of the paravascular retinal abnormalities has not been determined. Recent histopathological study showed that retinal cell debris was adherent to surgically removed internal limiting membranes (ILMs) in HM patients but not in non-HM patients^18^. Thus, pathological adhesions between the vitreous cortex and the ILM and inner retinal layer may play a role in the development of paravascular retinal abnormalities and vitreal changes.

The incidence of myopic RS has not been investigated in detail either at the macular area or paravascular area in young HM patients. The results of this study showed that myopic RS was found in 3 young HM eyes (1 child and 2 young adults), and the myopic RSs were paravascular RS and not macular RS. In addition, paravascular retinal cysts and vascular microfolds were seen more commonly in eyes with paravascular RS than those without. Our recent investigation using UWF-OCT also showed the relationship between myopic macular RS, paravascular vitreal adhesions and paravascular retinal cysts in adult HM patients^19^. Accordingly, it is possible that HM eyes with paravascular retinal cysts may be predisposed to paravascular RS and that the paravascular RS in young HM patients expands toward the macular area during aging and myopia progression. We conclude that the UWF-OCT is powerful tool for detection and following of paravascular RS in young HM patients by being able to examine a wide area of the fundus simultaneously.

**Figure 7:**
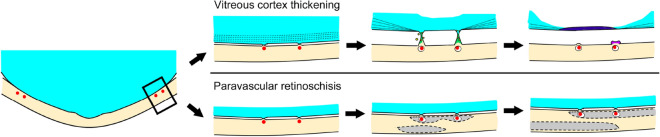
Schematic illustration showing the course of development of the vitreous cortex thickening and paravascular retinoschisis (RS). In the paravascular area, lamellar structural posterior vitreous cortex attaches to the inner retinal surface. As the posterior vitreous detachment progresses due to an irregular vitreoretinal adhesion, perivascular retinal tissue and posterior vitreous cortex pull each other. Then the perivascular retinal cells (yellow) migrate on the vitreous cortex layer and form bridging tissues (green). Finally, the dislocated perivascular cells form a lamellar vitreal cortex layers and merge into a single thick vitreous membrane (dark blue) and deteriorated perivascular tissues occasionally remain as debris on the retinal surface (purple). On the other hand, broad paravascular vitreal adhesions induce paravascular RS initially (grey). Finally, we suggest that the paravascular RS extends to the macular area and eventually develops a macular RS.

How and what kinds of paravascular changes occur between the vitreous and retina in young HM eyes are shown in Fig. 7. PVDs develop in accordance with irregular vitreoretinal adhesion at the site of retinal vessels. Paravascular retinal tissues are pulled anteriorly and the dislocated perivascular cells are forced into lamellar vitreal cortex layers to merge into a single thick vitreous membrane. On the other hand, broad paravascular vitreal adhesions occasionally induce paravascular RS. Paravascular RS extend toward macular area and eventually develops macular RS.

This study has several limitations. First, our study did not have a population-based recruitment of its participants. Thus, it is not clear whether the results can be directly transferred to other HM patients in the general population. The definition of HM for patients under 20 years-of-age was defined same as adult patients in our study. Thus, HM children and HM adolescent might be more myopic than those of general population. In addition, we divided the HM patients under 20 years-of-age into 2 groups of HM children and HM adolescent, and the classification might affect the results of our study. Second, this was not a longitudinal study, thus, it is not clear whether the paravascular RS actually progresses to macular RS with time. There were not many eyes with bridging tissues and paravascular RS, and we do not have strong evidence about high myopia and paravascular abnormalities in young HM patients. The progression of the vitreous cortex thickening and paravascular RS are assumed based on our findings. Third, due to retrospective nature of this study, we did not have sufficient non-HM adolescent and young adults. Accordingly comparisons of UWF OCT findings between HM and non-HM eyes have not been performed in adolescents and young adults. Then, earlier PVDs in HM adolescents and young adults were not examined in this study. However, we have shown earlier PVDs in patients who are > 50-years-old age using UWF OCT. Thus, we believe HM eyes develop a PVD earlier than non-HM eyes throughout adulthood.

In conclusion, posterior vitreous detachments occur in HM children but not in non-HM children, and the incidence increases with aging. Early partial PVD may play a role in the thickening of the vitreous cortex. An intense vitreoretinal traction with bridging tissues connecting the posterior vitreous and retinal vessels may cause various paravascular abnormalities accompanied by the migration of damaged tissues of the paravascular changes. These may initiate the pathological and proliferative vitreous changes in HM eyes. Paravascular RS is already present at an early age which may progress to macular RS with aging.
